# Large flexion contracture angle predicts tight extension gap during navigational posterior stabilized-type total knee arthroplasty with the pre-cut technique: a retrospective study

**DOI:** 10.1186/s12891-022-05035-z

**Published:** 2022-01-22

**Authors:** Takaaki Hiranaka, Shinichi Miyazawa, Takayuki Furumatsu, Yuya Kodama, Yusuke Kamatsuki, Shin Masuda, Yuki Okazaki, Keisuke Kintaka, Toshifumi Ozaki

**Affiliations:** Department of Orthopaedic Surgery, Okayama University Hospital, 2-5-1 Shikatacho, Kitaku, Okayama, 700-8558 Japan

**Keywords:** Total knee arthroplasty, Extension gap, Flexion gap, Predictor, Navigation system

## Abstract

**Background:**

This study aimed to determine the predictors of tight extension gap (EG) compared with the flexion gap (FG) during navigational posterior stabilized-type total knee arthroplasty using the pre-cut technique.

**Methods:**

Nineteen patients with tight EG (defined as FG-EG ≥2 mm after pre-cut; group T) and 84 patients with an approximately equal gap (defined as FG-EG = 0–1 mm after pre-cut; group E) were enrolled. Medial tibial slope angle, hip knee ankle angle, flexion contracture angle, and active maximum flexion angle were compared between the two groups.

**Results:**

The multivariate logistic regression model indicated that the probability of tight EG increased with flexion contracture angle (odds ratio, 1.13; 95% confidence interval 1.05–1.20; *P* ≤ 0.001). According to the receiver operating characteristic analysis, the flexion contracture angle cut-off value associated with tight EG was 15.0° (sensitivity, 85%; specificity, 78%).

**Conclusion:**

This study demonstrated that a large flexion contracture angle (cut-off 15.0°) was associated with tight EG after pre-cut osteotomy during posterior stabilized-type total knee arthroplasty. Awareness of this risk factor may help improve preoperative predictability of tight EGs and preparedness for additional procedures, such as soft tissue release or capsulotomy, to correct them.

**Level of evidence:**

Level III.

## Background

In total knee arthroplasty (TKA), implant alignment in both coronal and sagittal planes is crucial. Balanced flexion gap (FG) and extension gap (EG) are critical factors for a successful TKA because gap balancing influences final knee kinematics, postoperative range of motion, poorer functional scores, and increased rates of implant failures [1–4]. Measured resection and gap techniques have been used to make the equal “bone gap” in extension and flexion. However, even after adequate gap spaces were achieved using these techniques, the component setting can affect the EG because the posterior condylar of the femoral component can protrude posteriorly and tighten the posterior capsule. This phenomenon results in a “bone gap” and “component gap” mismatch [5, 6].

To prevent this mismatch, the pre-cut technique was advocated by Kaneyama et al. The recently introduced pre-cut technique involves cutting a portion of the posterior femoral condyle to create a small flexion space [7]. This approach facilitates the removal of the posterior osteophytes and the posterior soft tissue release through the temporary small flexion space. After resection of the medial and lateral menisci, posterior osteophytes, and release of soft tissue, which mainly influence EG, the additional posterior condylar cut thickness is determined based on the estimated gap with the pre-cut trial component attached. Therefore, the pre-cut technique helps create more accurate and balanced component gaps.

During posterior stabilized (PS)-type TKA, the posterior cruciate ligament is removed, which tends to widen the FG compared with the EG [8, 9]. Even after a pre-cut osteotomy is applied, the FG tends to be wider than the EG, especially in cases of flexion contracture deformities [10]. To achieve an equal FG and EG, a few selective EG expansion methods have been described [8, 11]. Kaneyama et al. reported that posteromedial vertical capsulotomy (PMVC), which involves cutting the joint capsule behind the medial collateral ligament, can be used to selectively expand the EG [12]. Masuda et al. reported that selective expansion of the EG by approximately 2.7 mm is possible by PMVC in PS-type TKA [13].

Although the importance of balanced EG-FG has been established, factors predicting a tight EG, compared with FG, in PS-type TKA, remain unclear. Several studies have reported that persistent flexion posture may lead to progressively increasing tightness of the collateral ligaments, posterior soft tissue, and hamstring muscles, which could be associated with a tight EG [14, 15]. Therefore, this study aimed to determine the predictors of a tight EG, compared with an FG, during navigational TKA surgery. It was hypothesized that a large flexion contracture angle (FCA) would be associated with an increased risk of tighter EG than FG ≥ 2 mm.

## Methods

The Internal Review Board of the concerned hospital approved this study. Each patient provided written informed consent. We studied 103 knees in 103 consecutive patients (21 male knees and 82 female knees) who underwent primary TKA using the Attune (DePuy-Johnson and Johnson, Warsaw, IN, USA) posterior stabilized (PS) component between January 2018 and December 2020. The exclusion criteria were revision TKA, primary TKA for posttraumatic osteoarthritis (OA), and insufficient navigation system evaluation data. All patients were diagnosed with primary osteoarthritis (*n* = 82) or rheumatoid arthritis (*n* = 21). Lower limb alignment was evaluated using the long leg standing X-ray preoperatively, and varus knees were recognized in 91 knees and valgus knees in 12 knees. All patients were retrospectively classified into two groups as follows: patients who had tight EG (FG – EG ≥ 2 mm) after pre-cut and needed the additional procedure, PMVC (group T, *n* = 19), and patients who had approximately equal (FG – EG = 0–1 mm) after pre-cut (group E, *n* = 84). All surgeries were performed by two senior authors (S.M. and Y.K.). The computer navigation program Vector Vision (CT-free, optoelectronic, passive marker navigation system, Brain-Lab, Munich, Germany) was used for all patients. After the precut-trial was set, the EG was measured at 0° and FG at 90° flexion using the navigation system. In addition, the differences between the EG and FG were evaluated. When the EG was smaller than the FG by ≥2 mm with the pre-cut trial set, PMVC was performed for expanding the EG selectively [7, 13]. Subsequently, the differences between the EG and FG were reevaluated after PMVC.

Patients’ demographics and clinical characteristics in each group are shown in Table 1. A goniometric measurement of the medial tibial slope (MTS) angle (MTSA) was performed on lateral radiographs by drawing two lines, as described previously [16, 17], defined by the longitudinal axis of the tibia and the MTS, respectively. The MTSA was defined as 90° minus the angle made by the intersection of the line of the longitudinal axis of the tibia and the MTS. FCA and active maximum flexion angle were evaluated using a goniometer (MMI goniometer, Muranaka Medical Instruments, Osaka, Japan) during the preoperative physical examination.

**Table 1:** Preoperative patients’ demographics and clinical characteristics

	Group T (***n*** = 19 patients)	Group E (***n*** = 84 patients)	***P*** value
Sex, male/female	4/15	17/67	n.s.^a^
Age, years	69.2 ± 7.9	71.6 ± 8.4	n.s.
Body mass index, kg/m^2^	26.7 ± 4.7	26.6 ± 4.2	n.s.
Diagnosis, osteoarthritis/rheumatoid arthritis	15/4	67/17	n.s.^a^
MTSA, °	9.0 ± 2.6	8.3 ± 3.2	n.s.
HKAA, °	10.4 ± 7.5	10.1 ± 5.0	n.s.
**FCA, °**	**18.2 ± 9.2**	**7.6 ± 7.6**	< 0.001*
Active maximum flexion angle, °	108.4 ± 25.5	120.5 ± 19.2	n.s.

Data are displayed as means ± standard deviation. Statistical differences were analyzed using the Mann–Whitney U test, except for Fisher’s exact tests (a). *MTSA* medial tibial slope angle, *HKAA* hip knee ankle angle, *FCA* flexion contracture angle, *n.s* not significant; (*) statistically significant (*P* <  0.05).

### Surgical procedure

**Fig. 1:**
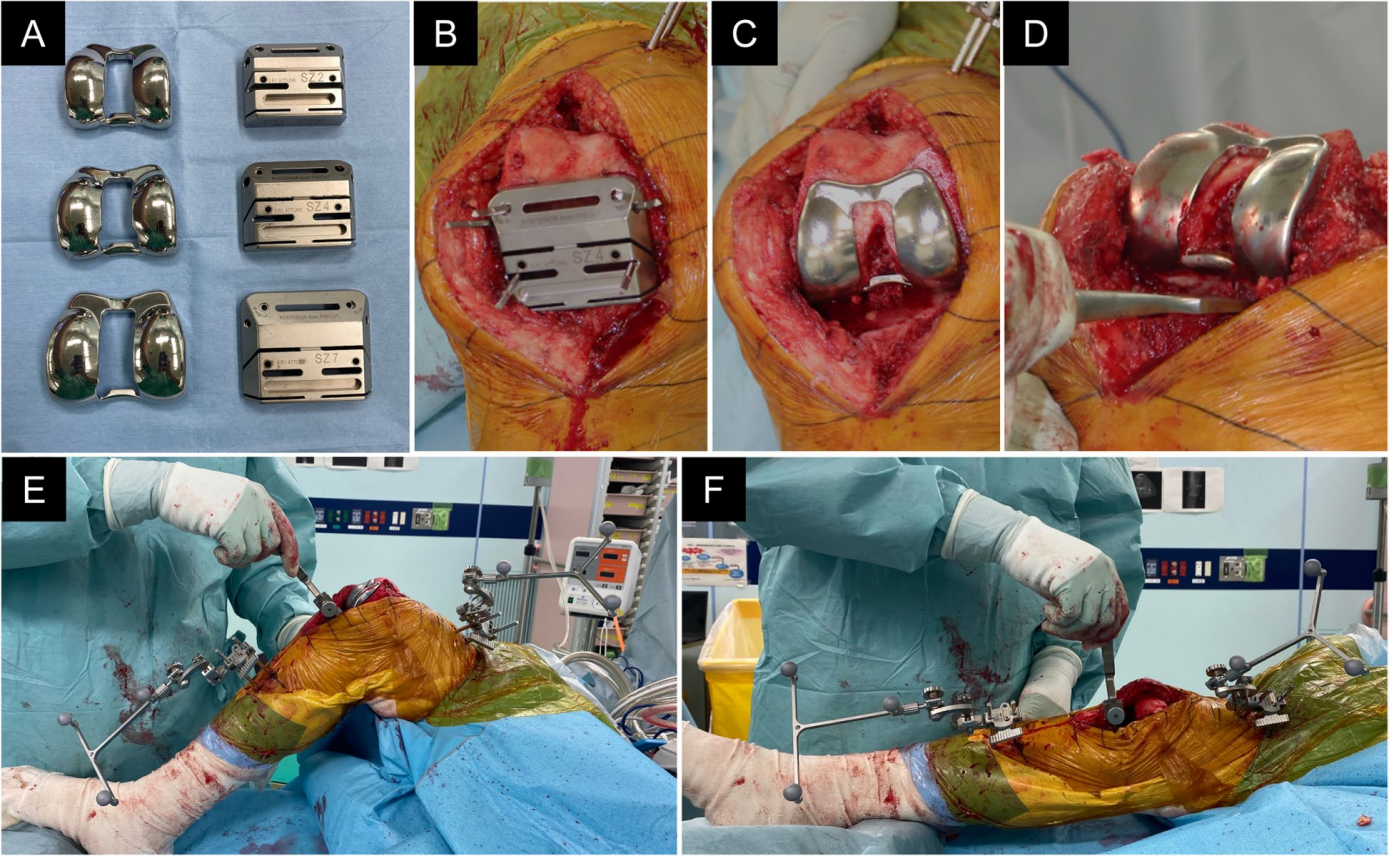
Intraoperative view of the pre-cut technique. **A** Appearance of the pre-cut trial component (left) and the cutting guide (right). Three sizes were available according to the patient’s bone size. **B**. A cutting guide for posterior femoral condylar pre-cut osteotomy. It enables surgeons to make a 4-mm pre-cut from the posterior femoral condylar axis. **C**. Pre-cut trial with a thickness of the portion of the posterior condyle 4 mm thinner than the ordinary implant. **D**. Removal of the posterior femoral condyle osteophytes. **E, F** With the pre-cut trial component inserted, the temporal FG (**E**) and EG (**F**) were measured using the navigation system at 90° knee flexion and knee extension. The spacer block was put in and out using two fingers smoothly with adequate tension and without lifting off. *EG* extension gap, *FG* flexion gap

The medial parapatellar and lateral approaches were used for varus knees and severe uncorrectable valgus knees, respectively. The pre-cut trial method was performed as reported previously [13]. First, the femoral distal cut was performed, followed by proximal tibial resection, which created the EG. Thereafter, the posterior femoral condyle was cut in two steps. A cutting guide for the posterior femoral condylar pre-cut was attached with a cutting plane, and a 4-mm pre-cut from the posterior femoral condylar axis was achieved (Fig. 1A, B). Further, it was attached in parallel with the surgical epicondylar axis (with a mean of 2.9° external rotation) from the posterior condyle axis under the indication of the navigation system. Afterward, a pre-cut trial with a thickness of the portion of the posterior condyle 4 mm thinner than the ordinary implant was prepared (Fig. 1C). Subsequently, medial and lateral meniscus and posterior femoral condyle osteophytes were removed to expand the EG (Fig. 1D). With the pre-cut trial inserted, the temporal FG and EG were measured using the navigation system at 90° knee flexion and knee extension using the spacer block (Fig. 1E, F). The measurement of the gap was performed by manual traction using a spacer block. In this procedure, the spacer block was put in and out using two fingers smoothly with adequate tension and without lifting off [18]. The FG and EG values were expressed as the value of the spacer block attached with the tibial base plate (9 mm). The measurement of the gap was performed by manual traction with appropriate tension and without lifting off. Basically, the gap evaluation was assessed by the medial gap, and lateral laxity was allowed within approximately 5° on average based on the previous reports demonstrating the physiological lateral laxity in normal knees [19, 20]. The appropriate size of the cutting guide and pre-cut trial were selected by the femoral sizer. The additional resection of the posterior femur was performed to set a femoral component to the distal bone. Additional resection of the posterior femur was 4 mm if the equal EG and FG were obtained and was 3 mm (1 mm undercut than usual) if the EG was smaller than FG by 1 mm. In addition, when the EG was smaller than the FG by ≥2 mm, PMVC was performed to expand the EG selectively, as reported previously [13]. For PMVC, first, a hole of approximately 1 cm was made in the posteromedial capsule at the distal lateral part of the medial collateral ligament using an electric scalpel. Second, the hole was expanded vertically from the medial femoral condyle to the medial tibial plateau using surgical scissors. Finally, the vertical hole was expanded using a spacer block. After the PMVC, the FG and EG were measured again.

### Statistical analysis

Statistical analysis was performed using EZR (Saitama Medical Center Jichi Medical University, Saitama, Japan) [21]. The Mann–Whitney U test was used to compare the values between the two groups. The statistical significance level was set at *P* <  0.05. A multivariate logistic regression analysis was performed for the values as risk factors for tight EG, which require PMVC. Separate univariate linear regression models were used to determine the association between the FCA and gap difference between flexion and extension. The FCA cut-off associated with increased possibility to be tight EG was determined using receiver operating characteristic (ROC) analysis and calculating the Youden index (J). The inter-observer and intra-observer reliabilities were assessed with the intra-class correlation coefficient (ICC). An ICC > 0.83 was considered a reliable measurement. To determine the interobserver reproducibility, the values of the EG and FG were investigated by two surgeons, and each surgeon evaluated each gap twice to calculate the intra-observer repeatability. The inter-observer reproducibility and intra-observer repeatability of the measurements of EG and FG were satisfactory when the respective mean ICC values were 0.88, 0.89, 0.92, and 0.94, respectively. Post hoc power analysis was performed using G*Power 3 (version 3.1.9.4; Heinrich Heine Universität Düsseldorf, DE). For a sample size of 19 in group T and 84 in group E and type-I error (α) of 0.05, the study was expected to provide a power (1 − β) of 0.80 for detecting an effect size of 0.45.

## Results

**Fig. 2:**
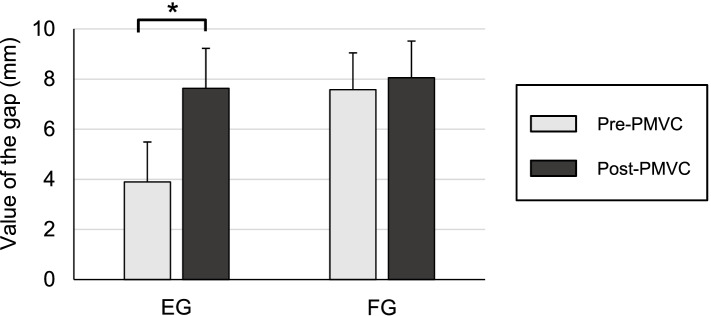
Gap values of the EG and FG pre- and post-PMVC in group T. The value of the EG in group T increased significantly after the PMVC (from 3.9 mm to 7.6 mm). However, the increase in FG was not significant (from 7.6 mm to 8.1 mm). *PMVC* posteromedial vertical capsulotomy, *EG* extension gap, *FG* flexion gap

The gap difference between flexion and extension after precut were significantly different between the two groups (average, group T = 3.7 mm; group E = 0.2 mm; *P* <  0.001). The value of the EG in group T significantly increased after the PMVC (from 3.9 mm to 7.6 mm); however, the FG was not increased significantly (from 7.6 mm to 8.1 mm) (Fig. 2). The gap difference between flexion and extension significantly decreased after PMVC by 3.3 mm from 3.7 mm to 0.4 mm in group T. There was no significant difference in the additional cut between the two groups (3.6 mm in group T and 3.8 mm in group E). Further, 4 mm additional cut was performed in 12 knees in group T and 18 knees in group E. Meanwhile, 3 mm additional cut (1 mm smaller than usual) was performed in seven knees in group T and 66 knees in group E (Table 2). The average final poly insert measured 8.4 ± 2.1 mm in group T and 8.0 ± 2.4 mm in group E. The final tibial slope was 3.4 ± 0.7° in group T and 3.2 ± 0.9° in group E. While the preoperative Insall–Salvati ratio was 0.89 ± 0.09 in group T and 0.90 ± 0.11 in group E, the postoperative Insall–Salvati ratio was 0.91 ± 0.07 in group T and 0.91 ± 0.08 in group E. No significant differences in insert thickness, tibial slope, and patellar height have been observed between the two groups.

**Table 2:** The gap difference between flexion and extension before and after PMVC

	Group T (***n*** = 19 patients)	Group E (***n*** = 84 patients)	***P*** value
**EG**	**3.9 ± 2.1**	**7.4 ± 1.7**	< 0.001*
**FG**	7.6 ± 1.5	7.6 ± 1.9	n.s.
**FG – EG**	**3.7 ± 1.7**	**0.2 ± 0.4**	< 0.001*
EG following PMVC	7.6 ± 1.6		
FG following PMVC	8.1 ± 1.5		
FG – EG following PMVC	0.4 ± 0.6		
Additional cut (4 mm/3 mm)	12 / 7	18 / 66	n.s.^a^

Data are displayed as means ± standard deviation. Statistical differences were analyzed using the Mann–Whitney U test, except for Fisher’s exact tests (a). *EG* extension gap, *FG* flexion gap, *PMVC* posteromedial vertical capsulotomy, *n.s* not significant; (*) statistically significant (*P* < 0.05).

The FCA (average, group T = 18.2°; group E = 7.6°; *P* <  0.001) was significantly different between the two groups (Table 1). There was no significant difference between the two groups regarding sex, age, body mass index, MTSA, hip knee ankle angle (HKAA), and active maximum flexion angle. The multivariate logistic regression model indicated that the odds of tight EG increased with FCA (odds ratio, 1.13; 95% confidence interval, 1.05–1.20; *P* ≤ 0.001). MTSA, HKAA, and active maximum flexion angle were not associated with an increased risk of tight EG (Table 3).

**Table 3:** Multivariate logistic regression analysis

Dependent variables	Significant variables	Odds ratio	P value	95% CI
Participants with or without tight EG, *n* = 103 patients	MTSA, °	1.060	0.560	0.88–1.27
	HKAA, °	0.976	0.617	0.89–1.07
	**FCA, °**	**1.130**	< 0.001*	**1.05–1.20**
	Active maximum flexion angle, °	0.992	0.571	0.96–1.02

The statistical analysis was performed using the forward stepwise method. *EG* extension gap, *MTSA* medial tibial slope angle, *HKAA* hip knee ankle angle, *FCA* flexion contracture angle, *CI* confidence interval; (*) statistically significant (*P* < 0.05).

**Fig. 3:**
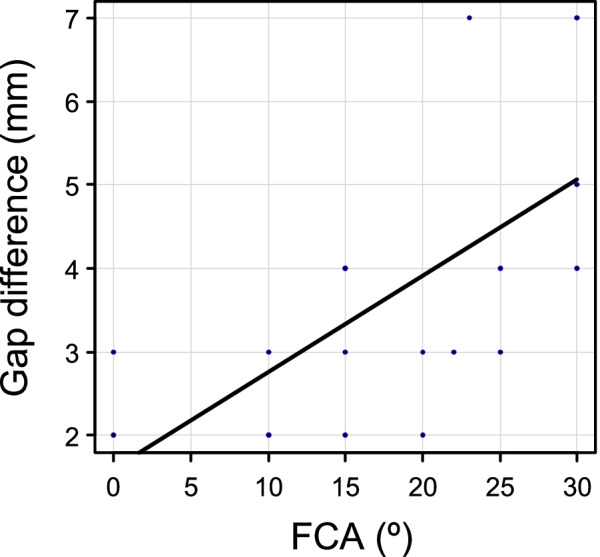
The correlation between the FCA and the gap difference between flexion and extension. The FCA and the gap difference between flexion and extension were significantly associated (R = 0.64; *P* = 0.003). *FCA* flexion contracture angle

**Fig. 4:**
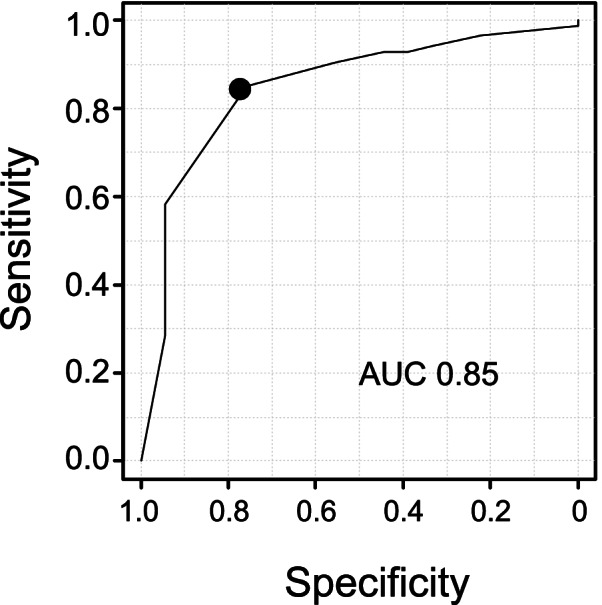
Threshold FCA for a tight EG. The calculated cut-off value (15.0°) had a sensitivity of 85% and a specificity of 78%. *AUC *area under the curve, *FCA* flexion contracture angle, *EG* extension gap

The FCA and the gap difference between flexion and extension were significantly associated, confirming that increased FCAs were correlated with a larger gap difference between flexion and extension (R = 0.64; *P* = 0.003) (Fig. 3). According to the ROC analysis, the FCA cut-off value associated with tight EG was 15.0°, with a sensitivity of 85% and specificity of 78%, and the calculated area under the curve was 0.85 (Fig. 4).

## Discussion

The most significant finding of this study was that large FCA (cut-off = 15.0°) was associated with a tight EG, as defined by an EG narrower than FG by ≥2 mm, after pre-cut osteotomy during PS-type TKA. These findings will help improve preoperative predictability of a tight EG, increasing preparedness for additional procedures, such as posterior soft tissue release or PMVC, to selectively expand the EG.

In this study, well-balanced EG and FG were achieved in a combined pre-cut technique and PMVC procedure. Preparing equal and rectangular extension and flexion joint gaps is the most critical goal in TKA because it facilitates functional restoration of the knee [22]. The pre-cut technique helps surgeons determine the additional need for bone resection based on the component gaps created in the pre-cut trial. The flexion gap space, created by the pre-cut of the posterior femoral condyle, facilitates posterior soft tissue release or osteophyte resection. The pre-cut technique can help perform additional posterior femoral condyle osteotomy while referring to the component gaps. In this study, a 1-mm undercut was allowed if the EG was smaller than FG by 1 mm. In addition, when the EG was smaller than the FG by ≥2 mm, PMVC was performed to selectively expand the EG without arranging for an additional bone resection [13]. However, the procedure involves the risk of anterior notch formation if a 2-mm undercut is performed as this system adopts the posterior reference, and the cutting line can shift by 2 mm posteriorly than planned. In this study, PMVC could selectively expand the EG by approximately 3 mm, and the final component gap difference between FG and EG was < 1 mm (Fig. 2 and Table 3). Based on these findings, we confirmed that the pre-cut technique along with PMVC can help achieve a well-balanced FG and EG.

We showed that a preoperative FCA of > 15° was associated with a tight EG. A persistent flexion posture caused by long-term inflammation eventually increased the tightness of collateral ligaments, posterior soft tissue, and hamstring muscles [15, 23, 24]. A possible reason behind this is a chronic tendency to assume the knee-mid-flexed position as a comfortable position and avoid painful extension. A previous study demonstrated that hamstring tightness was related to EG, and tight hamstring muscles may lead to a tight EG [14]. Patients with persistent flexion contractures have a normal FG but a narrow EG, and release of the posterior capsule and resection of posterior femoral condyle osteophytes increase the EG [25, 26]. The pre-cut technique facilitates removal of the medial and lateral menisci and posterior osteophytes; additional soft tissue management can be performed with limited resection of the posterior femoral condyles. In this study, larger preoperative FCA was associated with a tight EG even after resecting the posterior femoral condyle osteophytes using the pre-cut technique. In these cases, the tight EG was expanded selectively with PMVC by separating the tightened medial collateral ligament from the posterior capsule, achieving a well-balanced FG and EG. Besides, to selectively increase the EG, advanced planning for an additional distal femur osteotomy could be considered. However, additional distal femur osteotomy causes joint line elevation, which in turn causes altered patellofemoral kinematics, inferior clinical outcomes, limited knee range of motion, and midflexion joint laxity [27–30]. Thus, additional distal femur osteotomy should be minimal to avoid postoperative complications. In conclusion, preparations should be made for additional procedures, such as PMVC, if the preoperative FCA is > 15°.

Our study had a few limitations. First, this study was retrospective. Second, gap measurements were manually performed by a surgeon; these could not be used to evaluate tibiofemoral tightness. Potentially, a tensor device could have more accurately measured the gap to determine tibiofemoral joint tightness. Third, this study targeted only PS-type TKA, and the results may not be generalizable to other types of TKAs. Fourth, the femoral rotation angle during the pre-cut posterior femoral condyle osteotomy, which could affect the gaps, was not considered. Fifth, the postoperative clinical and radiographic outcomes, including knee range of motion, clinical scores, and lower limb alignment, were not evaluated because of the insufficient follow-up period in some patients, which is a significant study limitation. Therefore, further study with longer follow-up is warranted. Finally, patients with both varus and valgus knees were included in this study; however, there was no significant difference in patient demographics or gap sizes between the two groups in the sub-analysis.

The clinical relevance of this study is that an FCA over 15° could cause a tighter EG than FG (≥2 mm), which cannot be resolved by additional distal femoral osteotomy; however, surgeons should understand how to resolve this condition.

## Conclusion

This study demonstrated that larger FCA (> 15.0°) was associated with a tight EG following pre-cut osteotomy during PS-type TKA. Understanding these risk factors can help surgeons predict a tight EG preoperatively and prepare for additional procedures, such as PMVC, to expand the EG.

## Data Availability

The datasets used and/or analyzed during the current study are available from the corresponding author on reasonable request.

## Abbreviations

AUC: The area under the curve
CI: Confidence interval
EG: Extension gap
FCA: Flexion contracture angle
FG: Flexion gap
ICC: Intra-class correlation coefficient
MTS: Medial tibial slope
MTSA: Medial tibial slope angle
OR: Odds ratio
PMVC: Posteromedial vertical capsulotomy
PS: Posterior stabilized
ROC: Receiver operating characteristic
TKA: Total knee arthroplasty
